# Midazolam Ameliorates Acute Liver Injury Induced by Carbon Tetrachloride *via* Enhancing Nrf2 Signaling Pathway

**DOI:** 10.3389/fphar.2022.940137

**Published:** 2022-07-08

**Authors:** Yongyan Zhang, Yadi Zhu, Ying Li, Feng Ji, Guangbo Ge, Hua Xu

**Affiliations:** ^1^ Yueyang Hospital of Integrated Traditional Chinese and Western Medicine, Shanghai University of Traditional Chinese Medicine, Shanghai, China; ^2^ Shanghai Frontiers Science Center of TCM Chemical Biology, Institute of Interdisciplinary Integrative Medicine Research, Shanghai University of Traditional Chinese Medicine, Shanghai, China

**Keywords:** oxidative stress, Nrf2, midazolam, acute liver injury, molecular docking simulations

## Abstract

Oxidative stress contributes greatly to initiation and progression of liver injury. Activation of nuclear-factor erythroid 2-related factor 2 (Nrf2) has been considered as an attractive strategy for preventing and treating the oxidative damage related to liver injury. This study aimed to find an efficacious agent to activate Nrf2/HO-1 signaling pathway from clinically used therapeutic agents and to characterize the usefulness for preventing and treating CCl_4_-induced acute liver injury. For this purpose, a series of clinically used therapeutic agents were collected and their activation potentials on Nrf2 were assayed by using 293T-Nrf2-luc cell line. Among all tested therapeutic agents, midazolam was found with good Nrf2 activation effect and this agent could significantly ameliorate CCl_4_-induced damage to HepG2 cells. *In vivo* animal tests showed that pretreatment with midazolam reduced the liver pathological tissue damage and the serum levels of ALT and AST in CCl_4_-induced liver injury mice. Further investigations showed that midazolam could strongly up-regulate the expression of both Nrf2 and HO-1 in the mice liver, accompanied by increasing of the levels of antioxidant enzyme SOD and reducing the production of MDA, as well as reducing the pro-inflammatory cytokines (IL-6, TNF-α) secretion. Collectively, our results clearly demonstrate that midazolam can ameliorate CCl_4_-induced acute liver injury and oxidative stress *via* activating the Nrf2 signaling pathway.

## Introduction

Oxidative stress is a state of imbalance between oxidation and antioxidant effects in human body, which could result in the generation of a large number of reactive oxygen species (ROS) in cells. Under physiological conditions, excessive ROS will affect a series of cell transduction pathways, influence the production and secretion of oxidative stress molecules, and regulate the balance between proliferation and apoptosis of cells. Meanwhile, it can also affect the function of mitochondria, leading to cell dysfunction, and eventually damage various tissues and organs (Giustarini et al., 2009). Particularly, for perioperative and severe patients, oxidative stress can cause serious complications (e.g., inflammation, bleeding, postoperative cognitive dysfunction and organ dysfunction), whose incidence of these complications is as high as 30% (Toro-Pérez and Rodrigo, 2021).

More and more attention has been paid to the methods of reducing perioperative oxidative stress, such as endoscopic surgery (Arsalani-Zadeh et al., 2011), reducing the concentration of inhaled oxygen (Oldman et al., 2021) and giving antioxidant drugs (Ali- Hasan- Al- Saegh et al., 2016) and other methods. However, due to the limitations of surgical methods and patients’ own conditions, the clinical application of the first and second method are limited. The application of antioxidants signifies a rational curative strategy to prevent and cure liver diseases involving oxidative stress. Although conclusions drawn from clinical studies remain uncertain, animal studies have revealed the promising *in vivo* therapeutic effect of antioxidants on liver diseases. Particularly, some sedative and analgesic anesthetics have been widely concerned as treatment methods to reduce oxidative stress and protect important organ functions. Studies have shown that propofol can reduce the release of ROS and malondialdehyde (MDA) from the liver after ischemia-reperfusion, and contribute to the recovery of lung function (Chan et al., 2008). In the model of hepatic ischemia/reoxygenation, sufentanil increased superoxide dismutase (SOD), decreased MDA, reduced oxidative stress, and played a protective role in liver (Lian et al., 2019).

A large number of studies indicated that oxidative stress underlies the pathophysiology of various etiologies of liver disease, including chronic hepatitis C (Koike, 2007), alcoholic liver disease and non-alcoholic fatty liver disease (NAFLD)(Cederbaum et al., 2009), and contributes to the development of hepatocarcinogenesis (Cichoż-Lach and Michalak, 2014). Nuclear-factor erythroid 2-related factor 2 (Nrf2), known as the “main regulator” of antioxidant response, regulates a series of antioxidant and cytoprotective dependent protein genes by interacting with the antioxidant response element (ARE) sequences of antioxidant and cytoprotective genes (Kaspar et al., 2009), for example, heme oxygenase-1 (HO-1), quinone oxidoreductase1 (NQO1), glutathione-S-transferase (GST) (Tang et al., 2014). The enhanced activation of Nrf2 by pharmacologic molecules or genetic engineering has been shown to protect the liver in different oxidative stress models (Feng et al., 2021; Lim et al., 2021).

This study aimed to find drugs that effectively ameliorates acute liver injury through screen Nrf2 agonist from the commonly used analgesic. The selected positive Nrf2 agonist was applied to CCl_4_ liver injury model mice to determine its antioxidant and anti-inflammatory effects by detecting inflammatory factors and oxidative stress products. The results of this study can provide a new treatment strategy for perioperative and severe patients to reduce oxidative stress.

## Materials and Methods

### Construction of Stable Transfection Cells

293T-Nrf2-luc cell line was developed *via* lentiviral stable transfection of pGMLV-Nrf2-luc. pGMLV-Nrf2-luc is a kind of plasmid that designed by Genomeditech (Shanghai, China), which harbors Nrf2 promoter-luciferase reporter gene construct driven by multiple dioxin response elements. After that, 293T-Nrf2-luc cell line was obtained through antibiotic screening.

### Cell Culture

Both of 293T-Nrf2-luc cell lines and HepG2 cell lines were grown in DMEM/high glucose medium (Hyclone, United States) containing 10% fetal bovine serum (Hyclone, United States) and 1% penicillin-streptomycin solution (Hyclone, United States). The culture condition was constant temperature of 37°C, a humidified atmosphere of 95% air and 5% CO_2_. The fresh media were replaced every 2 days.

### Nrf2 Reporter Assay

293T-Nrf2-luc cell lines were first seeded in 96-well plates at a density of 8 × 10^3^ cells per well and grown overnight. Baicalin and iso-dihydromyricetin were prepared in our laboratory (Xiong et al., 2021; Zhao et al., 2021). Propofol (Fresenius Kabi, China), midazolam (Enhua, China), sufentanil (Humanwell, China), remifentanil (Humanwell, China), dexmedetomidine (Enhua, China), ketamine (Hengrui, China), dopamine (Fenghe, China),norepinephrine (Fenghe, China), epinephrine (Fenghe, China), diazepam (Xinyi, China), estazolam (Zhongxin, China), alprazolam (Enhua, China), rocuronium (Xianju, China), wogonin, liquiritin, anhydroicaritin, silybin A-SILYBIN-A, isoginkgetin, wogonoside, baicalein, scutellarein, scutellarin, hyperoside, liquiritigenin, glutamate, myricetin, dihydromyricetin, procyanidin (They were all purchased from Chengdu Purfield Biotechnology Co., Ltd. China), Baicalin and iso-dihydromyricetin were added at the final concentration of 10 μM, while sulforaphane (Sigma-Aldrich, United States) was used as a positive control (Russo et al., 2018). The negative control group was treated with the vehicle DMSO alone. The final concentration of DMSO in all experiments was below 1% (V/V).

Following 24 h incubation, luciferase activity was measured by the Steady-Glo^®^ Reagent (Promega, United States) according to the manufacturer’s directions. Luciferase activity was measured on Spectramax M3 (Molecular devices, United States) with luciferase production as readout. Data was shown as the percentage of fold induction of the test compound against the vehicle control group.

### Cell Viability Assay

HepG2 cells were seeded in 96-well culture plates (1.0  ×  10^4^ cells/well) and grown overnight. To verify the effects of midazolam on HepG2 viability, cells were treated with midazolam at different concentrations (0, 1.25, 2.5, 5, 10, 20, 40 μM) for 24 h. The cells were incubated with CCK-8 (DOJINDO Laboratories, Japan) solution (20 μl/well) and cultured at 37°C for another 1 h. The absorbance of the dissolved solutions was detected at 450 nm on Spectramax M3.

In order to verify the protective effect of midazolam on CCl_4_-induced HepG2 cells activity, different concentrations of CCl_4_ (0, 0.5, 1, 2, 4, 6%) were added to culture for 24 h, and HepG2 cells activity was detected by CCK-8 to determine the IC_50_ value of CCl_4_. After 24 h of intervention with different concentrations of midazolam, IC_50_ concentration of CCl_4_ was added for another 24 h of intervention, and finally CCK-8 was performed to detect cell activity.

### Animals Assay

The antioxidant effect of midazolam was tested in adult C57BL/6 mice (male, 6–8 weeks, 18–22 g) provided by Shanghai Slake Experimental Animal Co., Ltd. All mice were fed in the laboratory at a temperature of about 22°C and a relative humidity of 50%, with a light-dark cycle for 12 h. A 1-week acclimatization period was adopted before the experiment, during which the mice also had free access to food and water. All animal experiments were approved by the animal ethics committee of Yueyang Hospital of integrated traditional Chinese and Western Medicine Affiliated to Shanghai University of traditional Chinese medicine (ethics No.: YYLAC-2020-0801).

Next, 32 mice were randomly divided into the following groups (*n* = 8 in each group): control, CCl_4_ group (CCl_4_, Sinopharm Chemical Reagent, China), CCl_4_ plus midazolam 4 mg/kg/day (CCl_4_ + M4 group); CCl_4_ plus midazolam 8 mg/kg/day (CCl_4_ + M8 group). In the CCl_4_ group, mice were intraperitoneally (i.p.) injected with 0.5% (v/v) CCl_4_ (10 ml/kg, dissolved in corn oil). In the CCl_4_ + M4 group or CCl_4_ + M8 group, midazolam was respectively i. p. injected at 4 or 8 mg/kg for 3 days, and 0.5% CCl_4_ was i. p. injected at 2 h after the last dose of midazolam. Mice were sacrificed 24 h after CCl4 injection, and blood and liver samples were collected. A part of the liver was immediately fixed in 10% formaldehyde for histopathological observation, while the rest was frozen in liquid nitrogen and stored at −80°C for other experiments.

In order to explore the effect of midazolam on CCl_4_-induced lethality, 40 mice were used and randomly divided into the CCl_4_ group and CCl_4_ + midazolam group (*n* = 20). Midazolam was i. p. injected at 8 mg/kg for 3 days, and 50% CCl_4_ (2.6 ml/kg, dissolved in corn oil) were i.p. injected at 2 h after the last dose of midazolam. The mortality of mice in each group was recorded up to 36 h after CCl_4_ administration.

### Histological Examination of Liver Damage

The liver of each mouse (*n* = 8) was fixed in 10% neutral buffered formalin for 48 h and embedded in paraffin. Then 4 μm thick sections were cut and stained with hematoxylin and eosin (H&E) according to standard procedure. The samples were observed and images were obtained using an optical microscope (Olympus, Japan).

### Biochemical Analysis for Blood and Liver

Blood samples were centrifuged at 3000 × *g* for 10 min at 4°C and the serum was collected to measure the activity of aspartate aminotransferase (AST) and alanine transaminase (ALT), which were measured by kits (Nanjing Jiancheng Bioengineering Institute, China). Liver tissue homogenization was performed using tissue homogenizer (FSH-2A, XFK, China). The homogenates were centrifuged (1,000 × *g*, 15 min) at 4°C, and the obtained supernatant was used for subsequent measurement of MDA and SOD by kits (Nanjing Jiancheng Bioengineering Institute, China). The levels of TNF-α and IL-6 in the liver tissues were measured using ELISA kits according to the manufacturer’s instructions (Elabscience, China).

### Western Blot Assay

Total proteins were isolated from the liver tissues and cells using protein extraction kit (Beyotime Biotechnology, China). The cell nucleus proteins were extracted using Nuclear and Cytoplasmic Protein Extraction Kit (Beyotime Biotechnology, China). Protein concentrations were measured using BCA protein assay kit (Beyotime Biotechnology, China). Equal amounts of protein from each sample were subjected to 10% sodium dodecyl sulfate-polyacrylamide gel electrophoresis (SDS-PAGE), and the separated proteins were transferred to PVDF membranes. Then, the membranes were blocked with 1 × TBST containing 5% nonfat milk for 2 h at room temperature and continued to incubate with primary antibodies against mice Nrf2 (1:1000, Abcam, United Kingdom), HO-1 (1:1000, Abcam, United Kingdom), β-actin (1:1000, Beyotime Biotechnology, China) overnight at 4°C. Subsequently, the membranes were washed three times with TBST and incubated with secondary antibody at room temperature for 2 h. Blots were visualized using a hypersensitive chemiluminescence kit (Biotechnology, China). The results were quantified by densitometry using ImageJ software.

### Molecular Docking Simulations

The 3D structure of Kelch-like ECH-associated protein 1(Keap1) was downloaded from the Protein Data Bank (PDB Code:1ZGK) (Beamer et al., 2005). AutoDockTools 1.5.6 was applied to pre-process of coordinate files of the Keap1 and ligand by adding polar hydrogens, calculating Kollman charges and assigning AD4 atomic type. Docking site of Keap1 was situated at the kelch-like site (KS) as well as the functional site of Keap1 described by Beamer et al. (Beamer et al., 2005). The grid box was placed at KS, and the docking simulations was conducted by AutoDock Vina (1.1.2). Further analyses of the docking result was conducted by Discovery Studio Visualizer (BIOVIA Discovery Studio 2019; Dassault Systèmes, San Diego, United States).

### Statistical Analysis

The data were analyzed with GraphPad Prism 7.0 (San Diego, CA, United States) and presented as the means ± standard deviation (S.D.). One-way ANOVA analysis was used for differences between groups. The survival rate was analyzed by the log-rank test *p*-values less than 0.05 were considered statistically significant.

## Results

### Screening of Therapeutic Drugs on Nrf2 Activation

**FIGURE 1:**
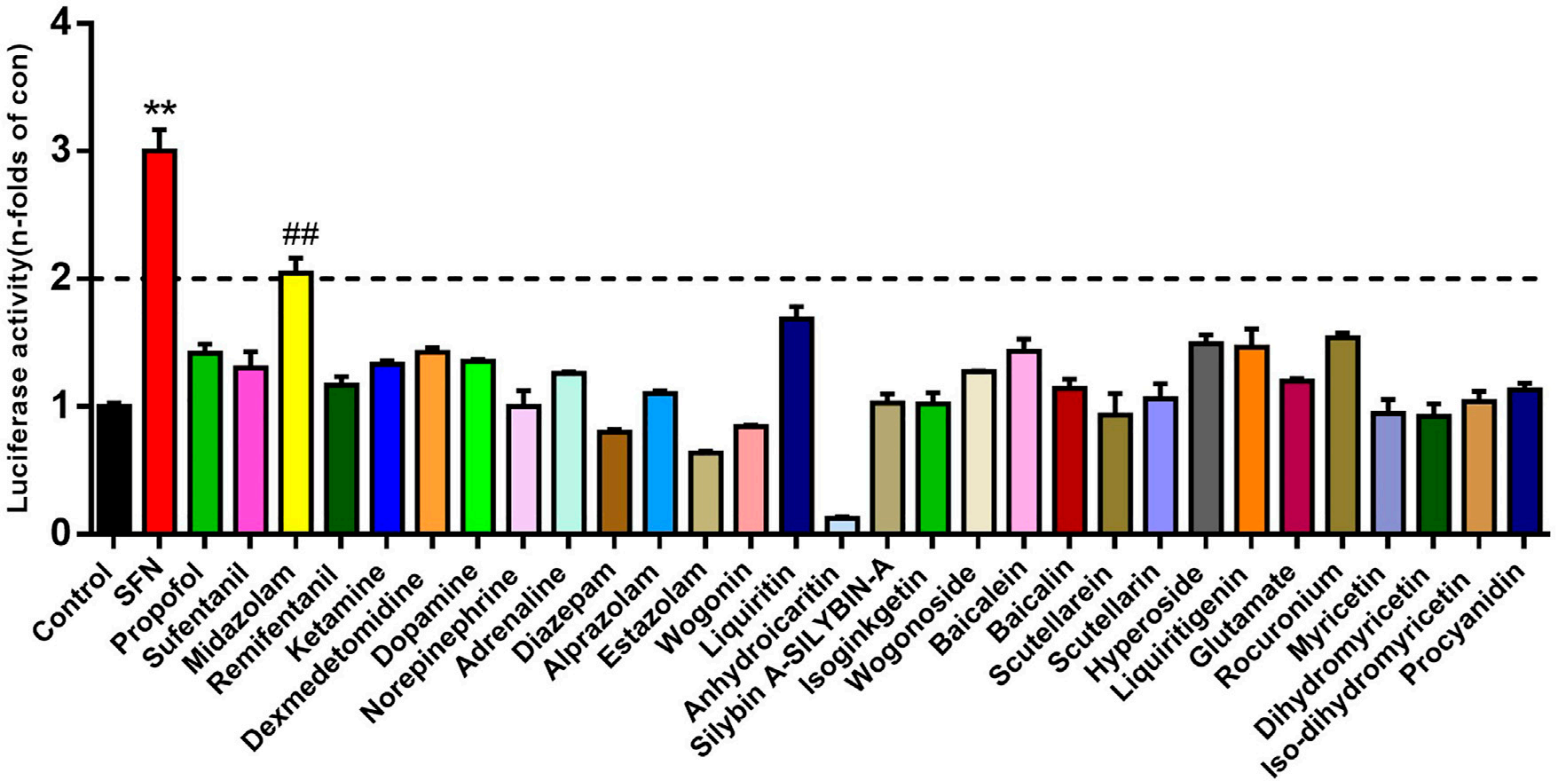
The activation effects of different therapeutic drugs on Nrf2. Luciferase report assay analyzed the activity of Nrf2 in 293T-Nrf2-luc cells after 24 h treatment with different therapeutic agents (10 μM, final concentration). Results were presented as the mean ± S.D. of six independent experiments. SFN, sulforaphane. The results were shown as mean ± S.D. **p < 0.01 vs. the control group; ##*p < 0.01* vs. the SFN group.

Using the Nrf2 luciferease reporter construct, the active effect of Nrf2 by 30 kinds of therapeutic agents (10 μM, final concentration) were evaluated. The results showed that midazolam up-regulated the activity of Nrf2 more than 2-fold compared with the negative control group *(p < 0.01)* (Figure 1).

### Midazolam Dose-Dependently Enhances Nrf2 Expression

**FIGURE 2:**
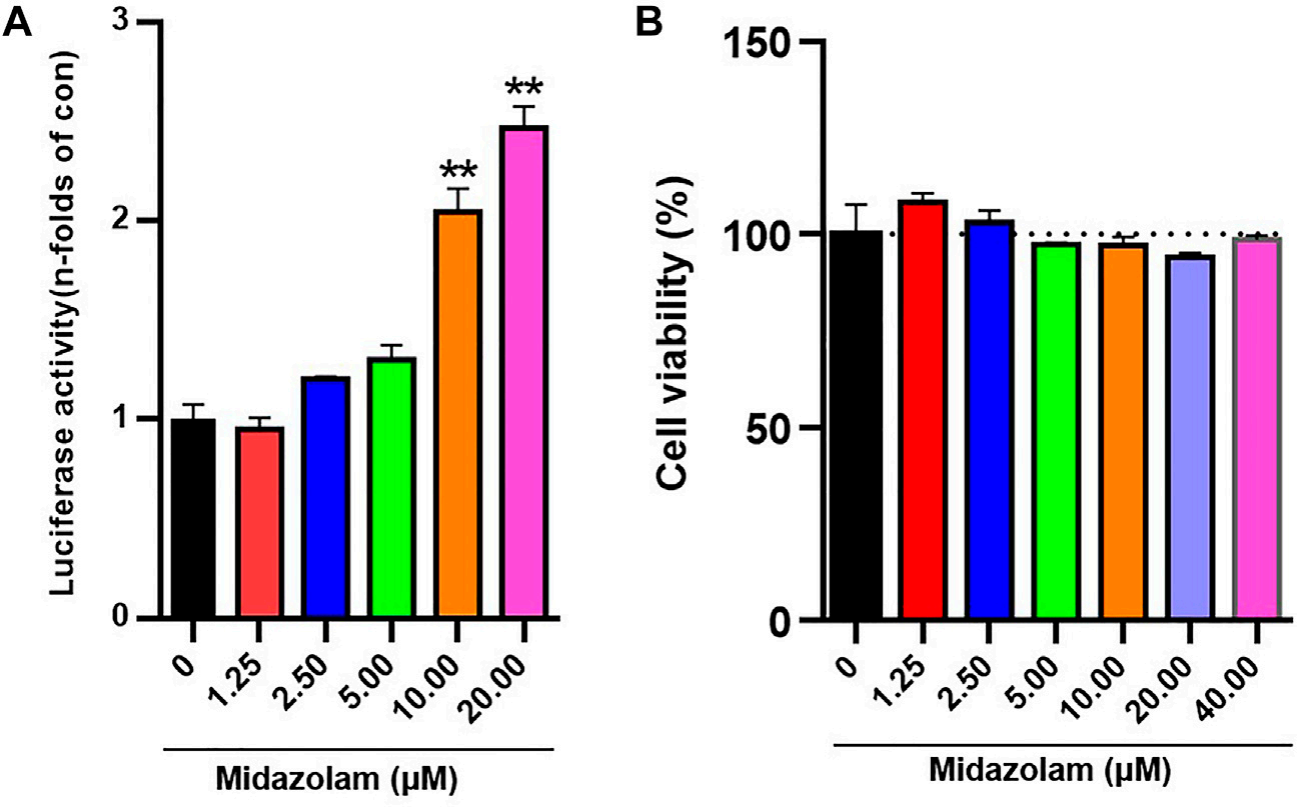
Effects of different concentrations of midazolam on Nrf2 activation and cell viability. **(A)** Luciferase report assay analyzed the activity of 293T-Nrf2-luc cells after 24 h treatment with different concentrations of midazolam (0, 1.25, 2.5, 5, 10, 20 μM); **(B)** CCK-8 assay analyzed the effect of different concentrations of midazolam (0, 1.25, 2.5, 5, 10, 20, 40 μM) on 293T-Nrf2-luc cells viability. The results were shown as mean ± S.D. **p < 0.01 vs. the control group.

The expression of Nrf2 activity in 293T-Nrf2-luc cells increased with increasing midazolam concentration, and was dose-dependent *(p < 0.01)* (Figure 2A). To determine the effect of midazolam on cell viability, cells were treated with different concentrations of midazolam for 24 h. Cell viability was measured by CCK-8 assay. As shown in Figure 2B, even if treatment with high-dose midazolam (40 μM), there was no significant difference compared with the control group.

**FIGURE 3:**
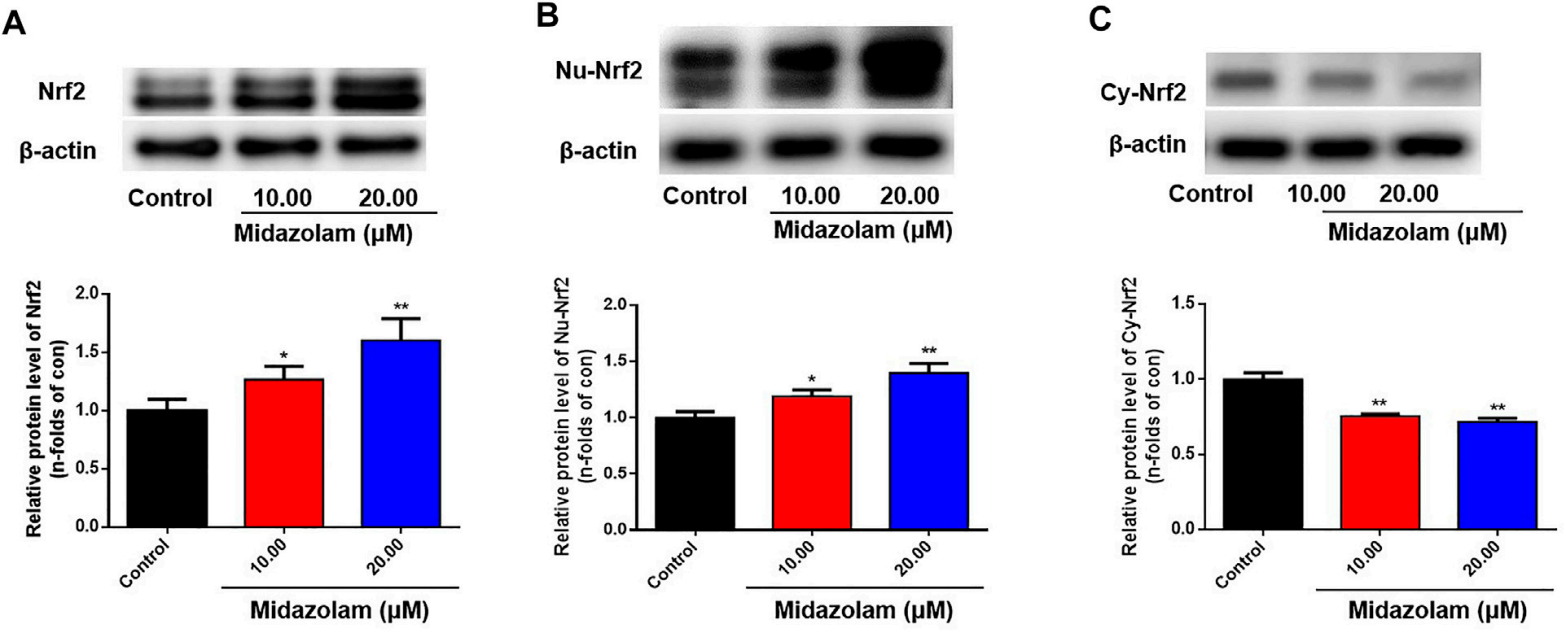
Effects of different concentrations of midazolam on Nrf2 protein level. 293T-Nrf2-luc cells were treated with different concentrations of midazolam (0, 10, 20 μM) for 24 h, and then using western blot to measure the level of Nrf2 protein. **(A)** Total Nrf2 protein level in cells; **(B)** Nrf2 protein level in the nucleus; **(C)** Nrf2 protein level in the cytoplasm. β-actin was used as an internal control for total protein. The results were shown as mean ± S.D. *p < 0.05 and **p < 0.01 vs. the control group.

To further verify the inductive effect of midazolam on Nrf2, the protein levels of Nrf2 in 293T-Nrf2-luc cells with or without midazolam treatment were determined. As shown in Figure 3A, Western blotting analysis showed that the protein expression of Nrf2 was elevated with the increasing concentrations of midazolam. It is well-known that Nrf2 is a transcriptional factor that needs to migrate to the nucleus to exert its function (Dinkova-Kostova et al., 2002). Under basal conditions Nrf2 is largely bound in the cytoplasm to Keap1, which is anchored to the actin cytoskeleton. When inducers disrupt the Keap1–Nrf2 complex, Nrf2 migrates to the nucleus where, in heterodimeric combinations with other transcription factors, it binds to antioxidant response element (ARE) regions of phase two genes and accelerates their transcription (Dinkova-Kostova et al., 2002). After the proteins in the nucleus and cytoplasm were extracted respectively, western blot analysis showed that with the increase of midazolam concentration, the Nrf2 protein level in the nucleus increased (Figure 3B) and the Nrf2 protein level in the cytoplasm decreased (Figure 3C). Midazolam promoted the translocation of Nrf2 to the nucleus.

### Midazolam Reduces the Damage of Hepatocytes Induced by CCl_4_

**FIGURE 4:**
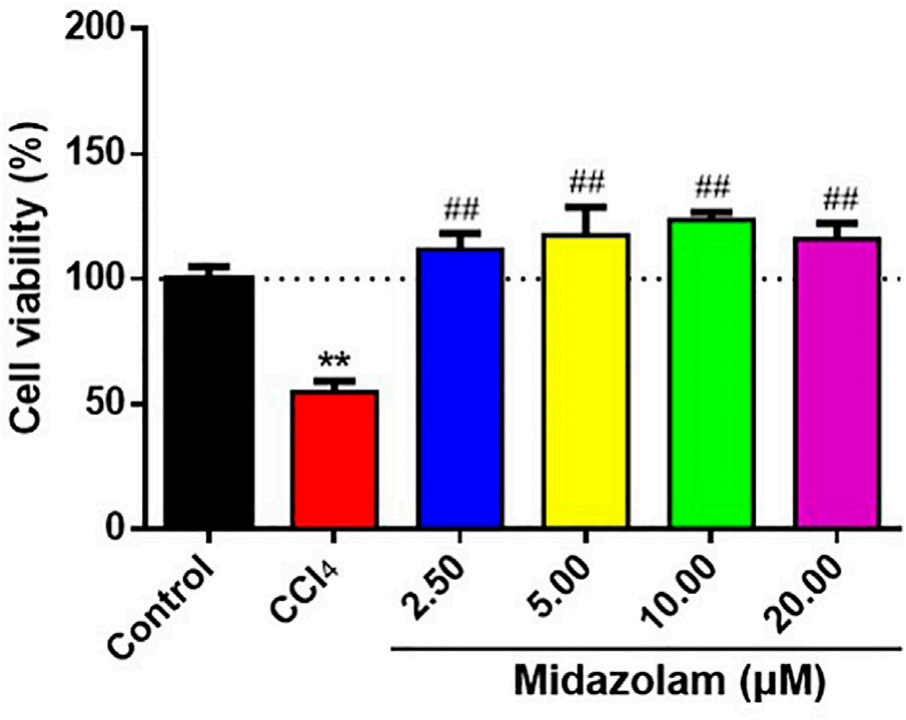
Effects of different concentrations of midazolam on HepG2 cells activity induced by CCl_4_. HepG2 cells were pretreated with different concentrations of midazolam (2.5, 5, 10, 20 Μm) for 24 h. CCl_4_ ((IC_50_ = 1.5%) induced HepG2 cells injury for 24 h. CCK-8 was used to analyze the effects of different concentrations of midazolam on cell viability induced by CCl_4_. The results were shown as mean ± S.D. **p < 0.01 vs. the control group; ##*p < 0.01* vs. the CCl_4_ group.

Next, the protective effects of midazolam on hepatocytes were investigated by using CCl_4_-induced HepG2 cells were used as model cells. After pretreatment of HepG2 cells with different concentrations of midazolam for 24 h, CCl_4_ (IC_50_ = 1.5%, Supplementary Figure S1) was added to induce cell damage. As shown in Figure 4, CCl_4_ significantly decreased HepG2 cells activity by more than 50% compared with the control group *(p < 0.01)*. By contrast, midazolam could significantly improve HepG2 cells activity compared with the CCl_4_ group (*p* < 0.01) even at low concentration (2.50 μM), as depicted in Figure 4. These findings clearly demonstrated that midazolam had good protective effects on CCl_4_-induced hepatocytes.

### Midazolam Attenuates CCl_4_-Induced Acute Liver Injury in Mice

**FIGURE 5:**
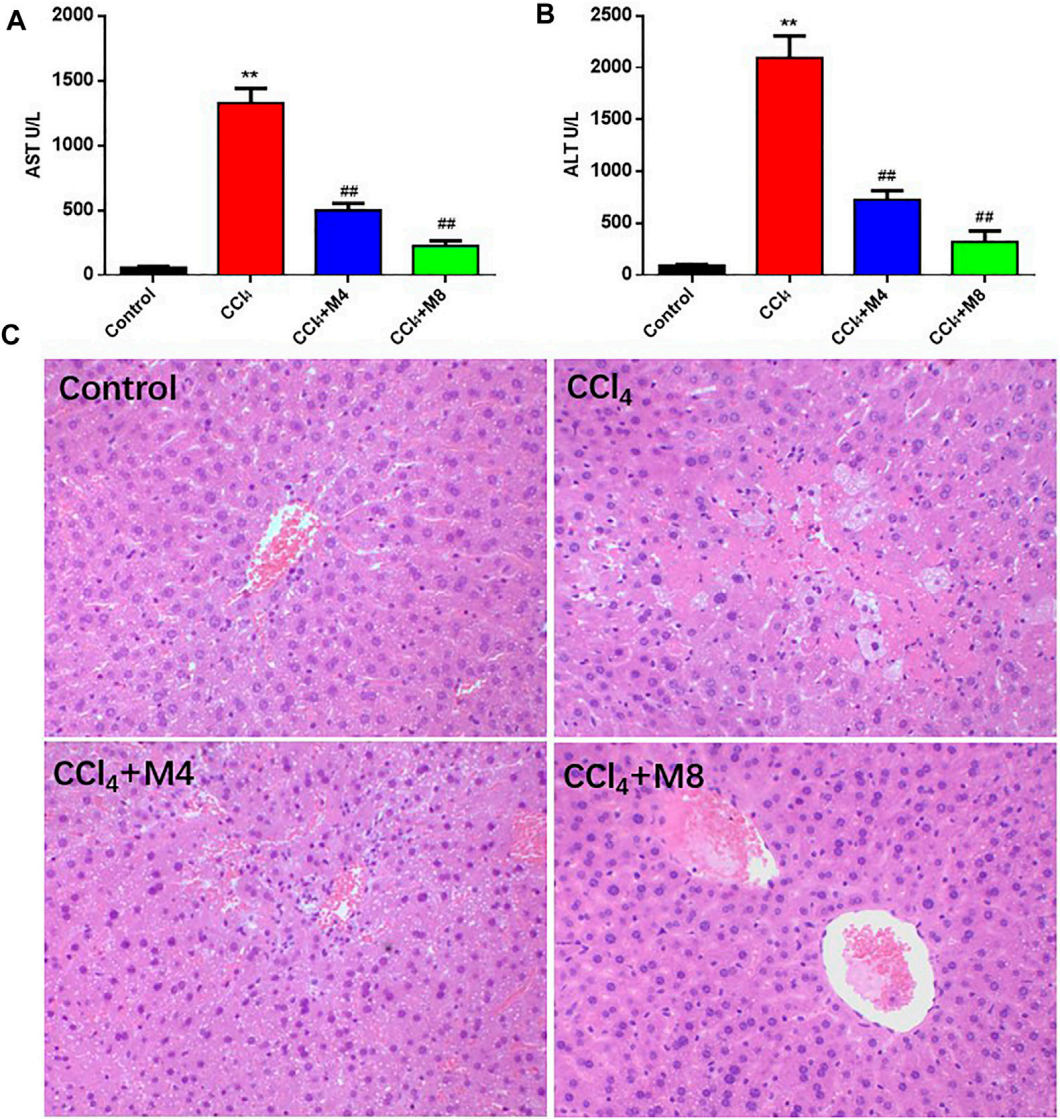
Protective effects of midazolam on CCl_4_-induced hepatic injury in mice. **(A**,**B)** The levels of AST and ALT in serum were detected at 24 h after CCl_4_ exposure. **(C)** Pathological images of H&E stained sections of liver in control, CCl_4_ and CCl_4_ plus midazolam treated mice (magnification, ×200). The results were shown as mean ± S.D. **p < 0.01 vs. the control group; ##*p < 0.01* vs. the CCl_4_ group.

Next, we evaluated the protective effects of midazolam (4 and 8 mg/kg/day, lasting for 3 days, i. p. injection) on CCl_4_ induced acute liver injury in mice. As shown in Figure 5, compared with the normal mice group, the activity levels of ALT and AST in serum of the CCl_4_ group were significantly increased (*p < 0.01*). In sharp contrast, the activity levels of ALT and AST in serum collected from midazolam pretreatment group were significantly decreased (*p* < 0.01), when compared with that from the CCl_4_ group (Figures 5A,B). Furthermore, H&E staining also suggested that the hepatocytes of the CCl_4_ group were swollen, while the liver cells were necrosis and a large number of inflammatory cells infiltrated. By contrast, following Midazolam pretreatment, the hepatocyte necrosis and the inflammatory cell infiltration were significantly reduced (Figure 5C). The histopathological scores showed a significant lower in both 4 and 8 mg/kg midazolam pretreatment compared to the CCl_4_ group (*p < 0.01*, Figure 2S). These findings clearly demonstrated that midazolam could attenuate CCl_4_ induced acute liver injury in mice.

### Midazolam Up-Regulates the Expression of Nrf2 and HO-1 in Mice Liver

**FIGURE 6:**
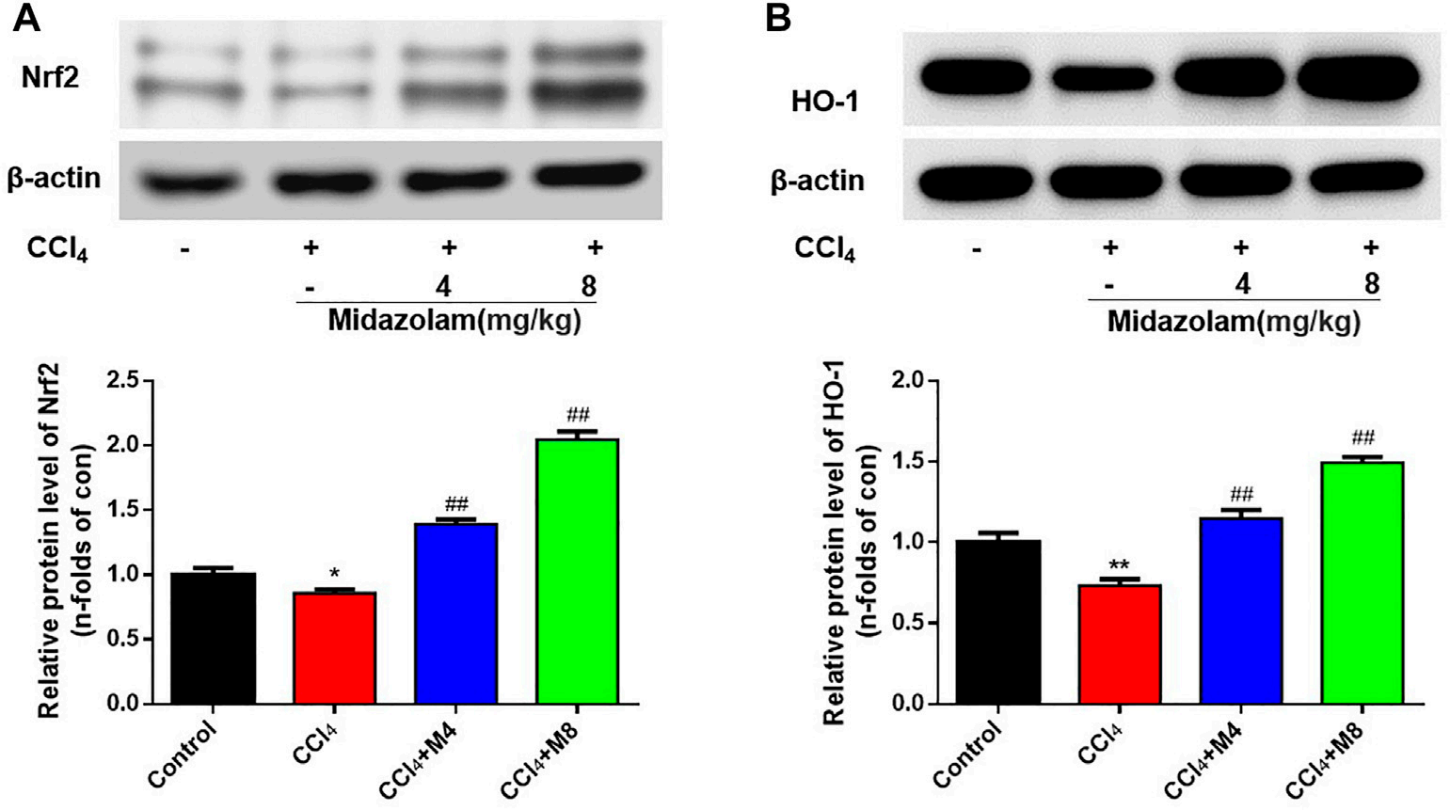
Effect of midazolam on the expression of Nrf2 and HO-1 in mice liver. The protein levels of Nrf2 **(A)** and HO-1 **(B)** were examined at 24 h after CCl_4_ exposure. (*n* = 8 in each group). **p < 0.01 vs. the control group; ##*p < 0.01* vs. the CCl_4_ group.

As shown in Figure 6, Western blot analysis showed that the expression levels of Nrf2 and HO-1 in the CCl_4_ group were down-regulated evidently when compared to that in the control group. However, following administration of midazolam, the protein levels of both Nrf2 and HO-1 were significantly enhanced. These findings clearly suggested that Nrf2-related signaling pathway in mice liver could be enhanced following administration of midazolam.

### Midazolam Ameliorates CCl_4_-Induced Oxidative Stress and Inflammation in Liver Tissue

**FIGURE 7:**
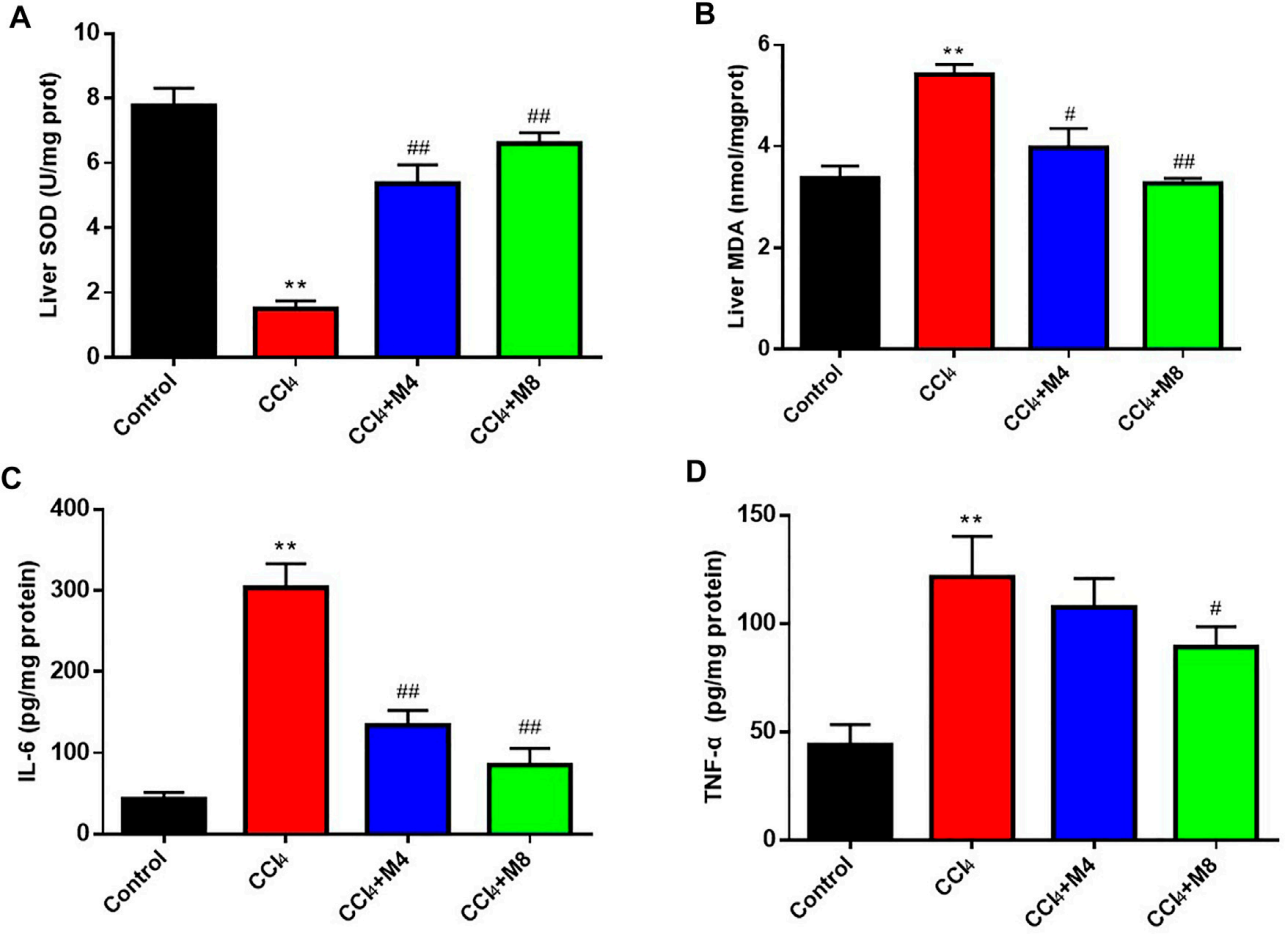
The effects of midazolam on the levels of SOD **(A)**, MDA **(B)**, IL-6 **(C)** and TNF-α **(D)** in mice liver. (*n* = 8 in each group). **p < 0.01 vs. the control group; #*p < 0.05* and ##*p < 0.01* vs. CCl_4_ group.

It is well-known that SOD and MDA are typical biomarkers of antioxidation and oxidative stress, respectively. As shown in Figure 7, compared with the control group, MDA levels in liver were increased *(p < 0.05)*, while SOD activity levels were decreased at 24 h after CCl_4_ treatment *(p < 0.05)*. Compared with CCl_4_ group, midazolam pretreatment group reduced MDA levels *(p < 0.05)* and increased SOD activity levels *(p < 0.05)* (Figures 7A,B). Meanwhile, it was also found that CCl_4_ treatment significantly increased TNF-α and IL-6 expression levels compared with the control group (*p < 0.01*). As expected, midazolam dose-dependently reduced IL-6 expression levels (*p < 0.01*) when compared with that in the CCl_4_ group, while the serum levels of TNF-α were also significantly reduced following administration with high dose midazolam (8 mg/kg) (*p < 0.05*) (Figures 7C,D).

### Midazolam Increases the Survival Rate of Mice Treated With Lethal Dose of CCl_4_

**FIGURE 8:**
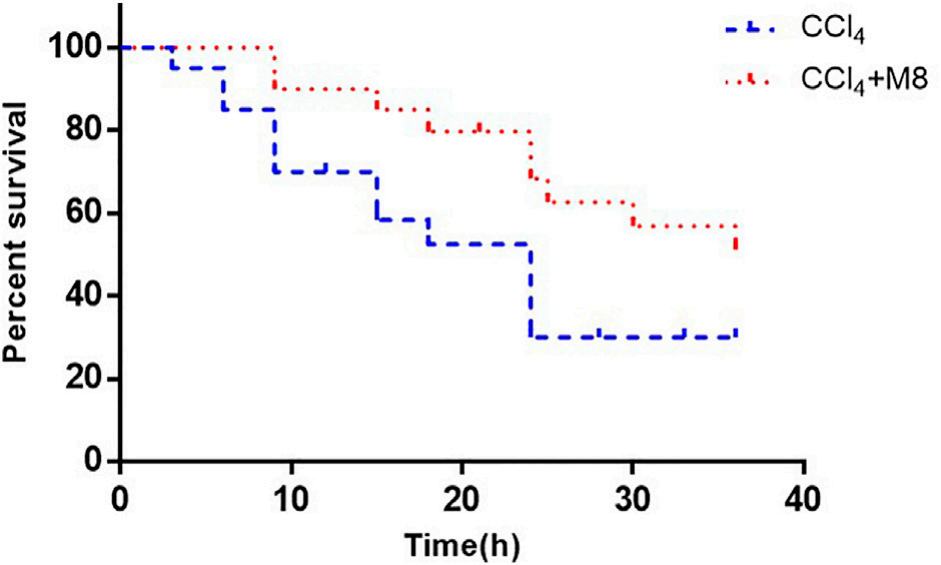
The effect of midazolam pretreatment on the survival rate of mice in which liver injury was induced by CCl_4_. Midazolam was i. p. injected at 8 mg/kg for 3 days 50% CCl_4_ (2.6 ml/kg) were i. p. injected at 2 h after the last dose of midazolam (*n* = 20). The results were analyzed using the log-rank test and expressed as Kaplan-Meier survival curves.

To further verify the hepatoprotective effect of midazolam, we investigated the ability of midazolam to affect the survival rate of mice treated with lethal dose of CCl_4_. For this purpose, a lethal dose of CCl_4_ for mice (2.6 ml/kg, 50%, i. p.) was used, while midazolam (8 mg/kg) was administrated for three consecutive days (once per day). As shown in Figure 8, midazolam significantly improved the survival rate of mice when compared with the CCl_4_ group (*p < 0.05*).

### Molecular Docking Simulations

**FIGURE 9:**
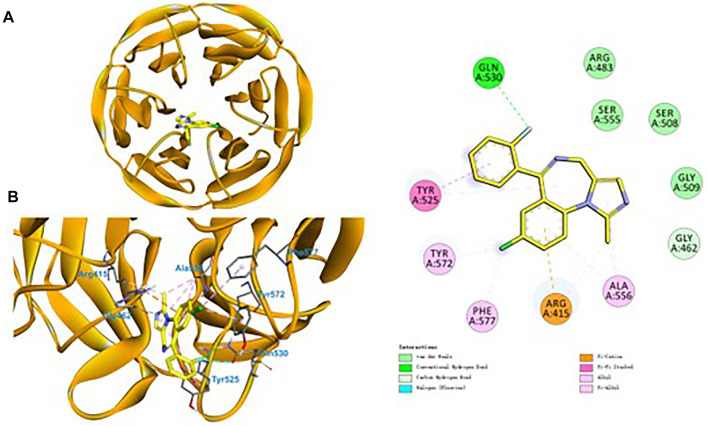
Ligand docking of midazolam in the kelch domain of human Keap1 protein.

Finally, to decipher the binding modes and the molecular mechanism of midazolam in enhancing of Nrf2 signaling pathway, molecular docking simulations of midazolam into Keap1 were performed. Under homeostatic conditions, Nrf2 is kept inactive being bound to its endogenous inhibitor Keap1 (Bellezza et al., 2018). As shown in Figure 9, midazolam could be well-docked into the kelch-like site (KS) of Keap1, forming strong hydrophobic interactions, electrostatic and hydrogen bonding interactions with several key residuals in the inner wall of KS. Remarkably, the unsaturated and aromatic functional groups of midazolam interacted with Ala556 and Tyr525 *via* strong hydrophobic interactions including Alkyl, Pi-Alkyl and Pi-Pi stacked interactions (Figure 9). It was particular that three phenyl containing lipophilic residues (Phe577, Tyr572 and Tyr525) stretched the side chains orienting the sheer center direction of KS, resulting a regional hydrophobic surface of KS inner wall (Figure 9). In addition, the group of chlorobenzene and fluorobenzene were vital for the binding of midazolam. The group of chlorobenzene interacted with Arg415 *via* Pi-Cation interactions, while the fluorobenzene could interact with Gln530 *via* hydrogen bonding. These observations clearly suggest that midazolam could stably and tightly bind to Keap1, which in turn, blocking the coalition between Keap1 and Nrf2.

## Discussion

Nrf2 is a key member of cap-n-collar basic leucine zipper transcription factor family, and is also a key regulator of mammalian genes involved in cell protection and detoxification by preventing oxidative stress (Martin et al., 1998). Under physiological conditions, Nrf2 mainly binds to Keap1 in cytoplasm (Yamamoto et al., 2018). After exposure to oxidative stress, Nrf2 and Keap1 are isolated and transferred to the nucleus. By interacting with the antioxidant response element (ARE) sequence of antioxidant and cytoprotective genes, Nrf2 induces the expression of a variety of antioxidant enzymes and phase II drug metabolism enzymes, including HO-1 and NQO1, and inhibits oxidative stress (Yamamoto et al., 2018)(Bellezza et al., 2018). Therefore, looking for Nrf2 activators has become a research hotspot in the prevention and treatment of oxidative stress-related diseases. Studies have shown that sedative and analgesic anesthetics commonly used in clinic can reduce the perioperative oxidative stress level. For example, in the acetaminophen induced liver injury animal model (Kostopanagiotou et al., 2009) and ischemia-reperfusion liver injury animal model (Chan et al., 2008), propofol can significantly reduce MDA, an index of oxidative stress in the liver. Another study has shown that ketamine can rapidly reduce the production of ROS and reduce protein damage, and play an antidepressant role in the hippocampus of ketamine treated mice through metabonomics and proteomics analysis (Weckmann et al., 2017). However, there is no comparative study on the effects of various common anesthetics on antioxidant stress. In our study, based on Nrf2 target, 30 kinds of therapeutic agents were screened by 293T-Nrf2-luc cell line to evaluate the inductive effect on Nrf2. The results showed that the inductive effect of midazolam on Nrf2 was more than 2 times, and the high dose of midazolam (20 μM) did not affect cell activity. This finding leads us to speculate that midazolam exerts stronger antioxidative stress ability than other anesthetics through Nrf2.

As the first step of metabolism, the liver is often exposed to high concentrations of exogenous and endogenous metabolites, especially vulnerable to oxidative damage caused by intermediate active substances. Nrf2/ARE, as one of the important mechanisms to protect the liver from oxidative stress, can play a protective role in liver inflammation, ischemia/reperfusion, fibrosis and regeneration by inducing target genes (Cuadrado et al., 2019; G; Bardallo et al., 2021). The activators of Nrf2, such as glycyrrhizic acid (Chen et al., 2013), sulforaphane (Wu et al., 2014), and curcumin (Peng et al., 2018), can induce the expression of antioxidant enzymes and reduce oxidative stress and liver injury. CCl_4_ is a strong hepatoxic and prooxidant agent widely used to induce hepatotoxicity, and its animal model has been widely used to screen the anti-hepatotoxicity and/or hepatoprotective activity of drugs (Yang et al., 2015; Cong et al., 2017; Zou et al., 2017). CCl_4_ causes a decrease in liver cells, in the activities of antioxidant enzymes and increases the protein carbonyl content, which is a protein oxidation product, at the oxidative stress biomarker MDA level (Sun et al., 2018). The purpose of this study was to evaluate the antioxidant capacity of midazolam *in vivo*, different doses of midazolam pretreatment were given to mice with acute liver injury induced by CCl_4_. Our results indicated that Midazolam pretreatment could significantly reduce the level of MDA which were markers of oxidative stress in liver tissue, and increased the activities of SOD antioxidant enzymes. Liver ALT, AST and pathological sections showed the protective effect of midazolam on liver. And we found that the higher the dose of midazolam, the greater the effect of reducing liver damage. Therefore, we chose a higher dose of midazolam to test the survival rate of mice given a lethal dose of CCl_4_. Midazolam significantly improved the survival rate of mice.

**FIGURE 10:**
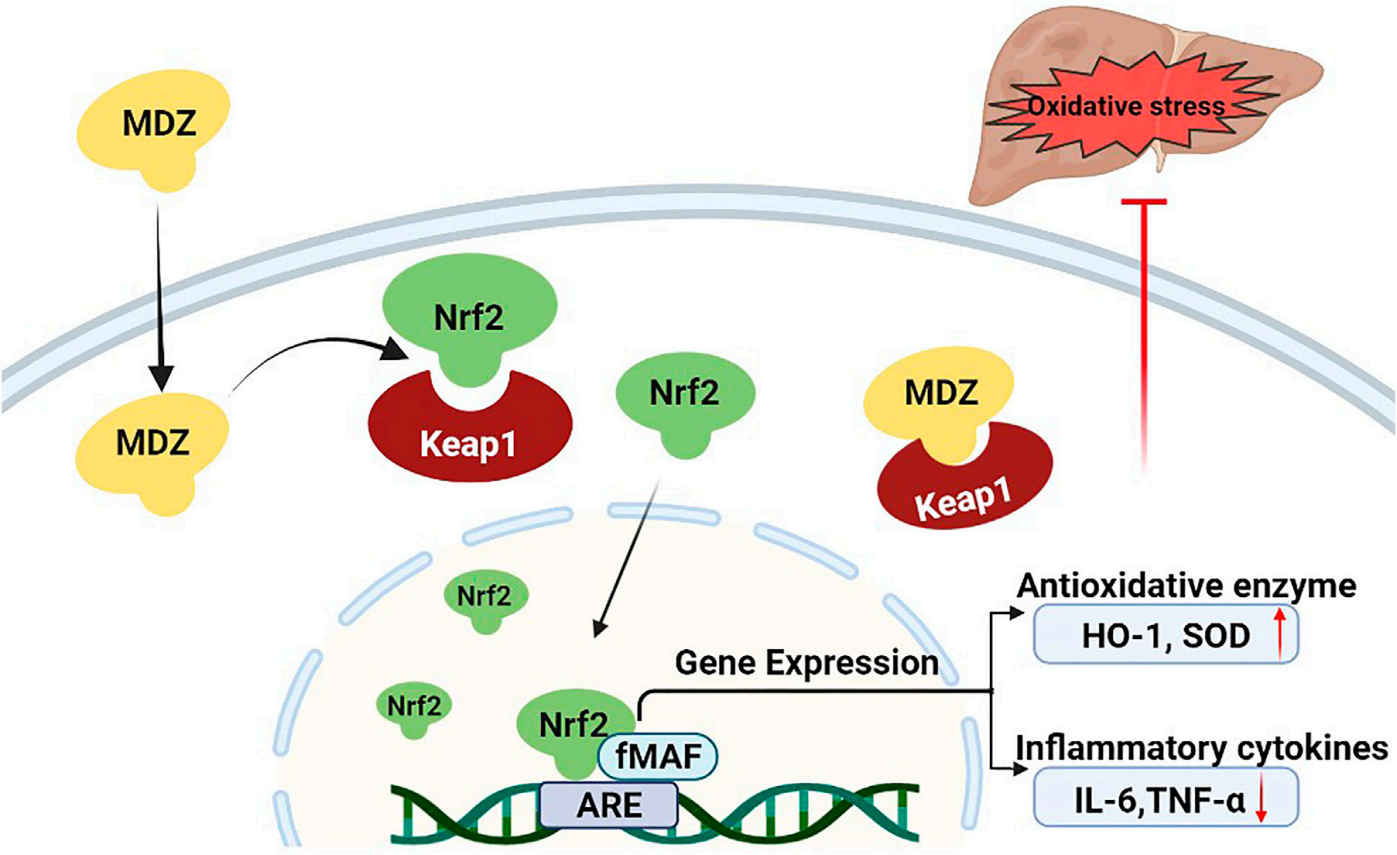
Proposed mechanism of midazolam for ameliorating acute liver injury and related oxidative stress induced by CCL_4_. Under normal physiological conditions, the Nrf2 protein level is tightly controlled by Keap1. Upon oxidative stress treatment, midazolam can bind to Keap1 stably, and hinder the coalition between Keap1 and Nrf2. Nrf2 is released and translocation to the nucleus to bind to AREs and initiate the anti-oxidative response by upregulating the expression of downstream target genes including HO-1 and SOD, which then decreases intracellular ROS production and alleviates oxidative stress-induced cytotoxicity. Midazolam ameliorates acute liver injury induced by carbon tetrachloride *via* enhancing Nrf2 signaling pathway.

Midazolam is a benzodiazepine sedative, which is commonly used in clinical anesthesia and intensive care unit (ICU) (Lazzaroni and Bianchi Porro, 2003; Garcia et al., 2021). Clinical research shows that the sedation of critical patients with midazolam 48 h continuous infusion can significantly reduce the level of IL-1β, IL-6 and TNF- α in blood (Helmy and Al-Attiyah, 2001). In LPS and galactosamine induced acute liver injury model mice, midazolam can reduce serum ALT and liver TNF-α levels, reduce liver injury and protect liver function (Li J. et al., 2018). Consistently, our results suggested that midazolam could also reduce the hepatic TNF-α and IL-6 induced by CCl_4._ Previous studies on the antioxidant stress of midazolam have mainly focused on the nervous system. Midazolam can inhibit oxidative stress, reduce the level of ROS, reduce the death of neurons, and play a neuroprotective role (Liu et al., 2017; Li GZ. et al., 2018). To conclude from the observation from molecular docking simulations, midazolam could bind to Keap1 stably, and hinder the coalition between Keap1 and Nrf2. This may be an important mechanism for midazolam to exert its anti-oxidative stress (Figure 10).

In summary, this study found that midazolam acted as an efficacious agent to activate Nrf2/HO-1 signaling pathway, which could enhance the levels of several key antioxidant proteins both *in vitro* and *in vivo*. Cell assays demonstrated that midazolam dose-dependently enhanced Nrf2 expression and showed good protective effects on CCl_4_-induced HepG2 cells. Animal tests showed that midazolam could significantly reduce the liver pathological tissue damage and the serum levels of both ALT and AST in CCl_4_-induced liver injury, accompanied by increasing of the levels of antioxidant enzyme SOD and reducing the production of MDA, as well as reducing the pro-inflammatory cytokines (IL-6, TNF-α) secretion. Molecular docking simulations suggested that midazolam could stably bind to Keap1 and hinder the binding of Keap1 to Nrf2. Collectively, our findings suggested that midazolam may be a good protector for alleviating CCl_4_-induced acute liver injury *via* enhancing Nrf2 signaling pathway, which offered a new pharmacotherapy for preventing and treating toxin-induced organ injury.

## Data Availability

The original contributions presented in the study are included in the article/Supplementary Material, further inquiries can be directed to the corresponding authors.

## Abbreviations

ALT, alanine transaminase; ARE, antioxidant response element; AST, aspartate aminotransferase; GST, glutathione-S-transferase; H&E, hematoxylin and eosin; HO-1, heme oxygenase-1; Keap1, Kelch-like ECH-associated protein 1; KS, kelch-like site; MDA, malondialdehyde; NAFLD, non-alcoholic fatty liver disease; NQO1, quinone oxidoreductase1; Nrf2, nuclear-factor erythroid 2-related factor 2; ROS, reactive oxygen species; SFN, sulforaphane; SOD, superoxide dismutase.
