# Supplementary figures and images for: Midazolam Ameliorates Acute Liver Injury Induced by Carbon Tetrachloride via Enhancing Nrf2 Signaling Pathway
Source: Front Pharmacol. 2022 Jul 8;13:940137. doi: 10.3389/fphar.2022.940137 (PMC9304748; doi:10.3389/fphar.2022.940137)

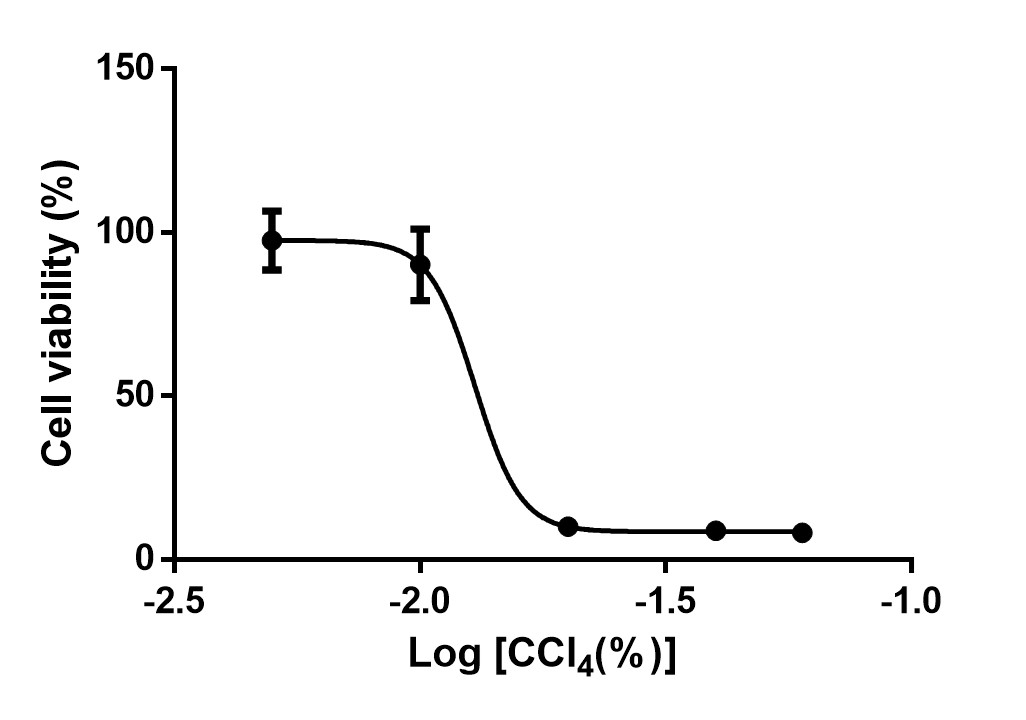

Supplement: Supplementary file 1 [file Image1.JPEG]

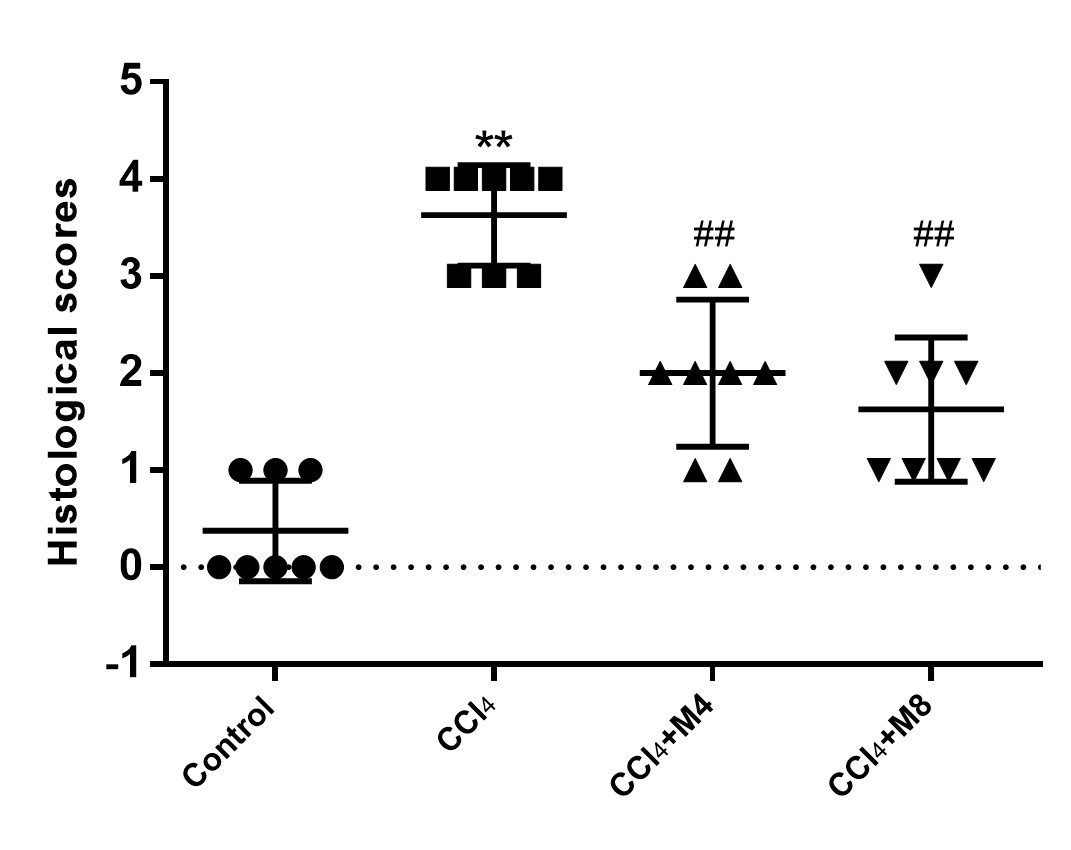

Supplement: Supplementary file 2 [file Image2.JPEG]
