# Supplementary material for: Midazolam Ameliorates Acute Liver Injury Induced by Carbon Tetrachloride via Enhancing Nrf2 Signaling Pathway
Source: Front Pharmacol. 2022 Jul 8;13:940137. doi: 10.3389/fphar.2022.940137 (PMC9304748; doi:10.3389/fphar.2022.940137)
Supplement: Supplementary file 3 [file DataSheet1.docx]

Supplementary Materials

**1. Determination of IC50 concentration for cellular injury of CCl_4_.**

Figure. S1 In order to verify the protective effect of midazolam on CCl_4_-induced HepG2 cells activity, different concentrations of CCl_4_ (0.5%, 1%, 2%, 4%, 6%) were added to culture for 24 hours, and HepG2 cells activity was detected by CCK-8 to determine the IC_50_ value of CCl_4_. The calculation results show that IC50 is 1.3%, so CCl_4_ of 1.5% is selected as the concentration in the experiment.

**2.The severity of necrotic lesions in the liver parenchyma.**

To evaluate the degree of necrosis after acute liver injury, an injury grading score (Grade 0–4) based on severity of necrotic lesions in the liver parenchyma were carried out as previously reported (Zhu et al., 2010). The scoring system was as follows: Grade 0, no pathological change; Grade 1, presence of degenerated hepatocytes with only rare foci of necrosis; Grade 2, small area of mild centrilobular necrosis around the central vein; Grade 3, area of mild centrilobular necrosis severer than Grade 2; and Grade 4, centrilobular necrosis severer than Grade 3. Each sample was independently scored by three pathologists who were not clear about the division of the treatment group and the untreated control group. It was showed that the histological score of CCl_4_ group was significantly higher than that of the control group (*p<0.01*). Compared with the CCl_4_ group, both 4 mg/kg and 8 mg/kg midazolam pretreated tissue scores were significantly lower (*p < 0.01*, Fig. 2S).

Figure. S2 the injury grading scores for liver sections (n = 8). The results were shown as mean ± S.D. ** p < 0.01 vs the control group; ## p < 0.01 vs the CCl_4_ group.

References:

Zhu R-Z, Xiang D, Xie C, *et al.* Protective effect of recombinant human IL-1Ra on CCl4-induced acute liver injury in mice. *World journal of gastroenterology* 2010; 16:2771–2779.
